# Corticosterone Facilitates Fluoxetine-Induced Neuronal Plasticity in the Hippocampus

**DOI:** 10.1371/journal.pone.0063662

**Published:** 2013-05-10

**Authors:** Katsunori Kobayashi, Yumiko Ikeda, Minoru Asada, Hirofumi Inagaki, Tomoyuki Kawada, Hidenori Suzuki

**Affiliations:** 1 Department of Pharmacology, Graduate School of Medicine, Nippon Medical School, Bunkyo-ku, Tokyo, Japan; 2 Japan Science and Technology Agency, Core Research for Evolutional Science and Technology, Saitama, Japan; 3 Department of Hygiene and Public Health, Graduate School of Medicine, Nippon Medical School, Bunkyo-ku, Tokyo, Japan; RIKEN Brain Science Institution, Japan

## Abstract

The hippocampal dentate gyrus has been implicated in a neuronal basis of antidepressant action. We have recently shown a distinct form of neuronal plasticity induced by the serotonergic antidepressant fluoxetine, that is, a reversal of maturation of the dentate granule cells in adult mice. This “dematuration” is induced in a large population of dentate neurons and maintained for at least one month after withdrawal of fluoxetine, suggesting long-lasting strong influence of dematuration on brain functioning. However, reliable induction of dematuration required doses of fluoxetine higher than suggested optimal doses for mice (10 to 18 mg/kg/day), which casts doubt on the clinical relevance of this effect. Since our previous studies were performed in naive mice, in the present study, we reexamined effects of fluoxetine using mice treated with chronic corticosterone that model neuroendocrine pathophysiology associated with depression. In corticosterone-treated mice, fluoxetine at 10 mg/kg/day downregulated expression of mature granule cell markers and attenuated strong frequency facilitation at the synapse formed by the granule cell axon mossy fiber, suggesting the induction of granule cell dematuration. In addition, fluoxetine caused marked enhancement of dopaminergic modulation at the mossy fiber synapse. In vehicle-treated mice, however, fluoxetine at this dose had no significant effects. The plasma level of fluoxetine was comparable to that in patients taking chronic fluoxetine, and corticosterone did not affect it. These results indicate that corticosterone facilitates fluoxetine-induced plastic changes in the dentate granule cells. Our finding may provide insight into neuronal mechanisms underlying enhanced responsiveness to antidepressant medication in certain pathological conditions.

## Introduction

Many animal studies have suggested that the dentate gyrus of the hippocampus is critically involved in mechanisms of action of antidepressant drugs including selective serotonin reuptake inhibitors (SSRIs) [1], [2], [3]. We have shown that the SSRI fluoxetine dose-dependently causes various effects on the mouse dentate granule cells and dentate-to-CA3 synaptic transmission mediated by the mossy fiber [4], [5], [6], [7]. Fluoxetine at 10 mg/kg/day can stabilize serotonin (5-hydroxytriptamine, 5-HT) 5-HT_4_ receptor-dependent modulation at the mossy fiber synapse by enhancing the effect of lower concentrations of serotonin and attenuating that of higher concentrations [4]. At higher doses, fluoxetine causes a strong enhancement of the serotonergic and D_1_-like receptor-dependent dopaminergic modulation at the mossy fiber synapse [5], [7]. In addition, high-dose fluoxetine causes “dematuration”, a reversal of the state of maturation, of adult dentate granule cells. The granule cell dematuration is characterized by suppression of mature physiological functions, reinstatement of high excitability of young neurons and reduced expression of molecular markers for mature granule cells [5]. One of characteristic functional properties of the mature granule cell is strong frequency facilitation, a form of presynaptic short-term synaptic plasticity, at the mossy fiber synapse [5], [8], and the granule cell dematuration causes a marked reduction of frequency facilitation to a juvenile level in adult mice [5]. These changes in synaptic transmission and modulation are maintained for at least one month after withdrawal of fluoxetine [6], [7], indicating a plastic nature of these effects. Therefore, the granule cell dematuration and enhanced monoaminergic modulations could be candidate neuronal processes underlying lasting effects of SSRIs in ameliorating symptoms of psychiatric disorders. However, these effects were not reliably induced at the suggested optimal dose range for mice (10 to 18 mg/kg/day) that gives rise to plasma drug levels comparable to those in patients taking chronic fluoxetine [9] and required higher doses of treatment, which raises a possibility that these are related to overdose effects of fluoxetine rather than therapeutic effects. It should be noted that our previous studies have been carried out using healthy intact mice. Responsiveness of granule cells to fluoxetine might be changed in pathological conditions. To test this possibility, in the present study, we reexamined effects of fluoxetine using mice chronically treated with the glucocorticoid corticosterone that model dysregulated hypothalamic-pituitary-adrenal (HPA) axis in major depression [10], [11], [12]. We found that chronic corticosterone facilitates effects of fluoxetine on the dentate granule cells without affecting plasma drug levels, and that fluoxetine at 10 mg/kg/day is sufficient for the robust enhancement of dopaminergic synaptic modulation and the induction of granule cell dematuration in corticosterone-treated mice.

## Materials and Methods

### Ethics Statement

All procedures were approved by the Animal Care and Use Committee of Nippon Medical School (Permit Number: 24-085).

### Drug Treatment

**Figure 1:**
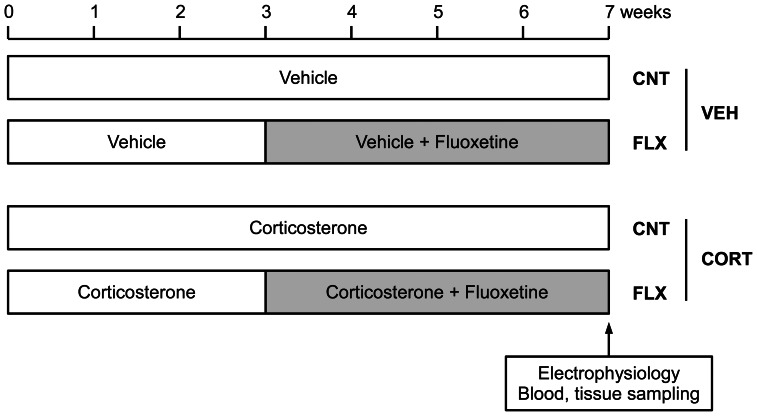
Schematic diagram showing timeline of corticosterone and fluoxetine administration. In control mice (CNT), corticosterone (CORT) or vehicle (VEH) was administered for 7 week. In fluoxetine-treated mice (FLX), fluoxetine was added during the last 4 weeks.

Male C57BL/6J mice were singly housed from the age of 8 weeks in the institutional standard condition (14∶10 light/dark cycle; lights on at 6∶00 A.M. through 8∶00 P.M.) at 23±1°C with food and water available ad libitum. Following 1 week of acclimation, mice were treated with corticosterone (Sigma-Aldrich, St. Louis, MO, USA) for 7 weeks at a dose of 10 mg/kg/day (Figure 1). Corticosterone was dissolved at a concentration of 2 mg/ml in distilled water containing a vehicle (2.8% hydroxypropyl-β-cyclodextrin, Sigma-Aldrich) and diluted in the drinking water. Concentrations of corticosterone in the drinking water were determined for individual mice everyday based on the water consumption during preceding 24 h and the body weight measured every other day. The vehicle solution was diluted and administered in the same way as the corticosterone solution. Fluoxetine hydrochloride (Wako Pure Chemical Industries, Ltd., Osaka, Japan) was added in the drinking water and administered at a dose of 10 mg/kg/day during the last 4 weeks of the treatment. In control mice, the vehicle or corticosterone treatment was continued (Figure 1).

### Measurement of Fluoxetine and Norfluoxetine in Plasma

Concentrations of fluoxetine and its metabolite norfluoxetine in mouse plasma were determined by the high-performance liquid chromatography (HPLC) according to the previous method [13]. Trunk blood was obtained at the time of brain dissection for the electrophysiological experiments and collected into a tube containing disodium ethylenediaminetetraacetic acid (EDTA) solution as anticoagulant. The tube was immediately centrifuged at 2700 g at 4°C for 10 min. The plasma sample was stored at −80°C until analysis. A 1.0-ml volume of 0.6 M sodium carbonate-sodium bicarbonate buffer (pH 9.8) containing the internal standard protriptyline (100 ng/ml) (Sigma-Aldrich) was added to 50 µl of plasma. After the addition of 7 ml of a mixture of ethyl acetate and n-heptane (20∶80, v/v), the mixture was vortexed for 1.5 min and then centrifuged at 3000 g for 10 min. The upper organic layer was transferred to another tube containing 0.2 ml of acidic phosphate buffer (0.025 M potassium dihydrogen phosphate adjusted to pH 2.3 with 85% phosphoric acid). The mixture was vortexed for 1 min and centrifuged at 3000 g for 10 min. The upper organic layer was removed by pipetting and further dried under a gentle stream of nitrogen for 10 min. Then, the residue of the aqueous phase was filtered through a 0.45-µm membrane filter. The filtrate was transferred to a tube for HPLC. The HPLC system composed of a pump with a 50-µl fixed volume autosampler and a UV detector (JASCO Corporation, Tokyo, Japan). The UV detector was set at 227 nm. The analytic column was Superspher 60 RP-8e (125×4 mm inside diameter, 4 µm particle size) with a cartridge guard column (4×4 mm inside diameter, 4 µm particle size) (Merck, Darmstadt, Germany). The mobile phase consisted of acidic aqueous solution (containing 0.1 ml of perchloric acid and 1.5 g of tetramethyl-ammonium perchlorate per liter) and acetonitrile (58∶42, v/v). The filtered mobile phase was used at a flow rate of 1.2 ml/min. The chromatographic run time was 15 min. Retention times were between 6.7 and 7.5 minutes for the internal standard, between 8.0 and 9.2 minutes for norfluoxetine and 10.3 and 11.9 minutes for fluoxetine. To prepare standard curves, appropriate amounts of fluoxetine and norfluoxetine were added to 50 µl of control plasma to yield 1 µg/ml. This sample was prepared according to the procedure as above. Quantification was performed by calculating the peak-height ratios of fluoxetine and norfluoxetine to the internal standard using software (JASCO Corporation, Tokyo, Japan).

### Electrophysiology

Mice were decapitated under deep halothane anesthesia and hippocampi were isolated. Transverse hippocampal slices (380 µm) were cut using a tissue slicer and electrophysiological recordings were performed as described [5], [14]. Recordings were made in a submersion-type chamber maintained at 27.0–27.5°C and superfused at 2 ml/min with saline composed of (in mM): NaCl, 125; KCl, 2.5; NaH_2_PO_4_, 1.0; NaHCO_3_, 26.2; glucose, 11; CaCl_2_, 2.5; MgCl_2_, 1.3 (equilibrated with 95% O_2_/5% CO_2_). Field excitatory postsynaptic potentials (fEPSPs) arising from the mossy fiber synapses were evoked by stimulating the dentate granule cell layer and recorded from the stratum lucidum of CA3 using a glass pipette filled with 2 M NaCl. The amplitude of fEPSPs was measured on analysis as described [14]. A criterion used to identify the mossy fiber input was more than 85% block of the fEPSP amplitude by an agonist of group II metabotropic glutamate receptors, (2S,2′R,3′R)-2-(2′,3′-dicarboxycyclopropyl)glycine (DCG-IV, 1 µM) (Tocris Bioscience, Bristol, UK). Single electrical stimulation was delivered at a frequency of 0.05 Hz for baseline recordings. Dopamine hydrochloride was purchased from Wako Pure Chemical Industries, Ltd. Serotonin hydrochloride was from Sigma-Aldrich. SKF81297 and SCH23390 were from Tocris Bioscience. All recordings were made using a Multiclamp 700B amplifier (Molecular Devices, Sunnyvale, CA, USA), filtered at 2 kHz and stored in a personal computer via an interface (digitized at 10 kHz).

### Quantitative RT-PCR Analysis

The hippocampal slices were prepared as in the electrophysiological experiments. The dentate gyrus was dissected out of the slice under a dissecting microscope and used for reverse transcription-polymerase chain reaction (RT-PCR) analyses. Nucleospin® RNA XS (TAKARA, Otsu, Shiga, Japan) was used to extract total RNA. cDNA was synthesized using PrimeScript® RT Master Mix (TAKARA). Quantitative PCR was performed using gene specific primers and PowerSYBR® Green PCR master mix (Applied Biosystems), using the StepOnePlus® real-time PCR instrument. The followings were primer sequences used. Calbindin, 5′- TCTGGCTTCATTTCGACGCTG and 5′-ACAAAGGATTTCATTTCCGGTGA; desmoplakin, 5′-GCTGAAGAACACTCTAGCCCA and 5′-ACTGCTGTTTCCTCTGAGACA; tryptophan 2,3-dioxygenase (TDO), 5′- ATGAGTGGGTGCCCGTTTG and 5′- GGCTCTGTTTACACCAGTTTGAG; β-actin, 5′-AGTGTGACGTTGACATCCGTA and 5′- GCCAGAGCAGTAATCTCCTTCT. PCR was carried out for 45 cycles (94°C for 15 s, 65°C for 1 m), followed by a melt curve (60°C to 95°C with 0.3°C step every 15 s). All data were normalized to β-actin and relative expression changes between conditions were calculated by the comparative CT method.

### Statistics

All data are presented as means ± SEMs. The number of data (n) represents the number of mice unless otherwise specified. Statistical significance was assessed by two-way ANOVA followed by the Bonferroni posttest with the significance level *P*<0.05. Statistical tests were performed using GraphPad Prism version 5.03 for Windows (GraphPad Software, La Jolla, CA, USA).

## Results

**Figure 2:**
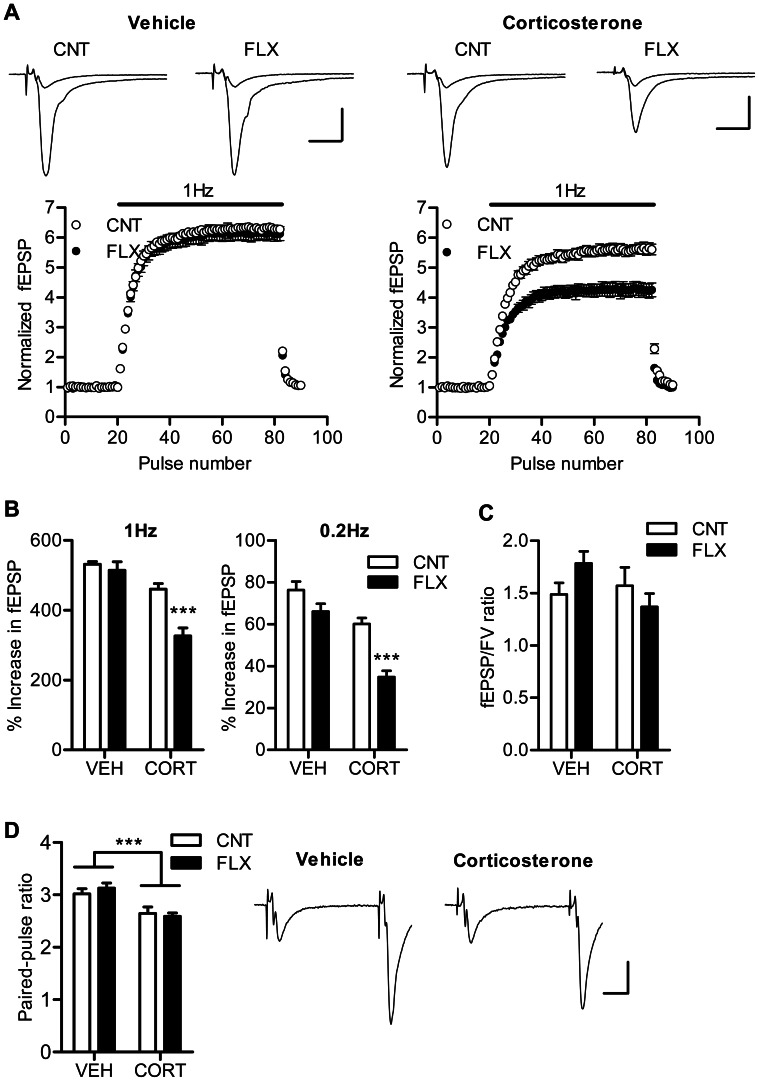
Corticosterone facilitates effects of fluoxetine on frequency facilitation. (A) The time course of frequency facilitation induced by 1-Hz stimulation. Sample traces show averages of 15 consecutive fEPSPs during baseline and 1 Hz stimulation. Scale bar: 10 ms, 0.5 mV. (B) Pooled data showing facilitated effects of fluoxetine on frequency facilitation at 1 Hz (CORT effect: *P*<0.0001, FLX effect: *P* = 0.0008, CORT×FLX: *P* = 0.0062, *n* = 6 to 7) and 0.2 Hz (CORT effect: *P*<0.0001, FLX effect: *P*<0.0001, CORT×FLX: *P* = 0.0361, *n* = 5 to 7) in corticosterone-treated mice. ***P<0.001 compared with CNT/CORT. (C) Lack of changes in ratios of fEPSP to presynaptic fiber volley (FV) amplitude (*n* = 6 to 7). (D) Reduced synaptic facilitation induced by paired stimulation at 50-ms interval in corticosterone-treated mice (*n* = 6 to 7). CORT effect: ***P = 0.0001. Sample traces are from CNT/VEH and CNT/CORT groups. Scale bar: 10 ms, 0.2 mV.

Mice were treated with corticosterone at a dose of 10 mg/kg/day for 7 weeks and also with fluoxetine at 10 mg/kg/day during the last 4 weeks (Figure 1). We measured plasma fluoxetine concentrations at the end of the treatment, and found no significant difference in the plasma level of fluoxetine or its metabolite norfluoxetine between corticosterone- and vehicle-treated mice (Table 1). Using this treatment regimen, we first examined the effect of fluoxetine on the hippocampal synaptic transmission in the absence and presence of corticosterone. Our previous study in naive mice has shown that chronic fluoxetine strongly suppresses frequency facilitation at the mossy fiber synapse only at higher doses (>18 mg/kg/day). Consistently, fluoxetine at 10 mg/kg/day had no significant effects on frequency facilitation induced by repetitive stimulation at 1 Hz or 0.2 Hz in the vehicle-treated mice (Figure 2A and 2B). In the corticosterone-treated mice, however, the same dose of fluoxetine significantly reduced the magnitude of facilitation (*P*<0.001), and there was significant interaction between corticosterone and fluoxetine treatments (1 Hz: *P* = 0.0062, 0.2 Hz: *P* = 0.0361) (Figure 2A and 2B). The corticosterone treatment itself appeared to have a suppressive effect on frequency facilitation. In addition, corticosterone significantly reduced paired-pulse facilitation, another form of short-term plasticity, as well, whereas fluoxetine did not affect it (Figure 2D). These decreases in synaptic facilitation could be due to an increase in probability of transmitter release from presynaptic terminals. However, neither corticosterone nor fluoxetine affected the ratio of fEPSP to presynaptic fiber volley amplitude (Figure 2C), suggesting a lack of effects of these treatments on the basal synaptic efficacy. These results indicate that corticosterone treatment can facilitate the effect of fluoxetine on frequency facilitation at the mossy fiber synapse and suggest that the phenotype of the mossy fiber synapse was altered by the fluoxetine treatment.

**Table 1:** Plasma fluoxetine and norfluoxetine levels (ng/ml).

	Vehicle (*n* = 5)	Corticosterone (*n* = 6)
Fluoxetine	648.6±39.9	645.4±46.9
Norfluoxetine	1118±71.3	1301±140.7

**Figure 3:**
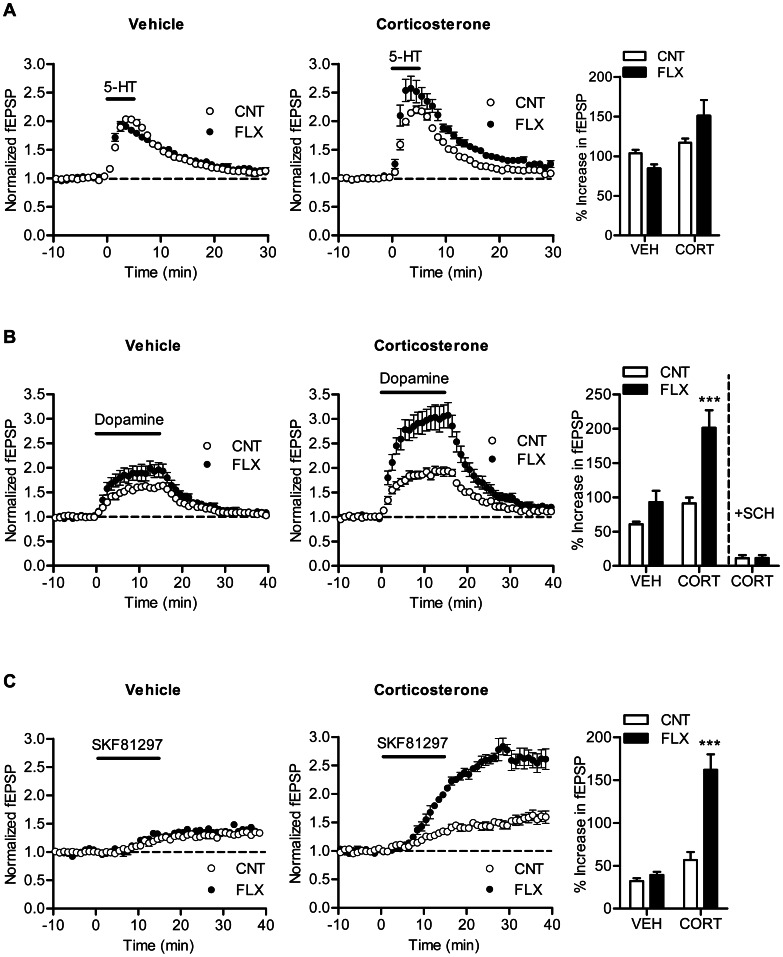
Effects of fluoxetine on monoaminergic synaptic modulation in corticosterone-treated mice. (A) Effects of fluoxetine on serotonin-induced synaptic potentiation. CORT effect: *P* = 0.0049, CORT×FLX: *P* = 0.0489 (*n* = 5 to 7). (B) Facilitated effects of fluoxetine on synaptic potentiation induced by dopamine (10 µM). CORT effect: *P* = 0.0006, FLX effect: *P* = 0.0004, CORT×FLX: *P* = 0.0336 (*n* = 6 to 7). ***P<0.001 compared with CNT/CORT. The bar graph at right includes the results from slices pretreated with SCH23390 (*n* = 4 slices each). (C) Facilitated effects of fluoxetine on synaptic potentiation induced by SKF81297 (100 nM). CORT effect: *P*<0.0001, FLX effect: *P* = 0.0004, CORT×FLX: *P* = 0.0011 (*n* = 3 to 4 slices). ***P<0.001 compared with CNT/CORT.

Mossy fiber synaptic transmission can be potentiated by dopamine [14] and serotonin [4]. Chronic fluoxetine enhances the dopaminergic synaptic modulation [7] and either suppresses or enhances the serotonergic modulation in a dose-dependent manner [4], [5]. Next we examined effects of fluoxetine on these monoaminergic synaptic modulations in corticosterone-treated mice. Fluoxetine slightly reduced synaptic potentiation induced by 5-µM serotonin in vehicle-treated mice, which is consistent with our previously results observed in a similar experimental condition [4]. In corticosterone-treated mice, however, fluoxetine caused a small enhancement of serotonin-induced synaptic potentiation on average, and there was significant interaction between corticosterone and fluoxetine treatments (*P* = 0.0489) (Figure 3A), suggesting that fluoxetine differentially affects the serotonergic modulation in the presence and absence of corticosterone. This tendency is similar to the dose-dependent switch in the direction of effects of fluoxetine on the serotonergic modulation [4], [5]. Corticosterone also changed the effect of fluoxetine on the dopaminergic modulation. Fluoxetine caused a significant enhancement of dopamine-induced synaptic potentiation in corticosterone-treated mice (*P*<0.001), but not in vehicle-treated mice (Figure 3B). There was significant interaction between corticosterone and fluoxetine treatments (*P* = 0.0336), indicating that corticosterone facilitates the enhancement of the dopaminergic modulation by fluoxetine. In naive mice, the dopaminergic modulation at the mossy fiber synapse is mediated by D_1_-like receptors and nearly completely suppressed by the D_1_-like receptor antagonist SCH23390 [14]. In corticosterone-treated mice, the dopamine-induced synaptic potentiation was strongly attenuated by pretreatment of hippocampal slices with SCH23390 (30 nM) in both control and fluoxetine-treated groups (Figure 3B). The D_1_-like receptor agonist SKF81297 can induce slowly developing synaptic potentiation at the mossy fiber synapse [14]. This SKF81297-induced synaptic potentiation was enhanced by fluoxetine in corticosterone-treated mice (*P*<0.001), but not in vehicle-treated mice (Figure 3C). Thus, corticosterone facilitated the effect of fluoxetine on the D_1_-like receptor-dependent synaptic potentiation.

**Figure 4:**
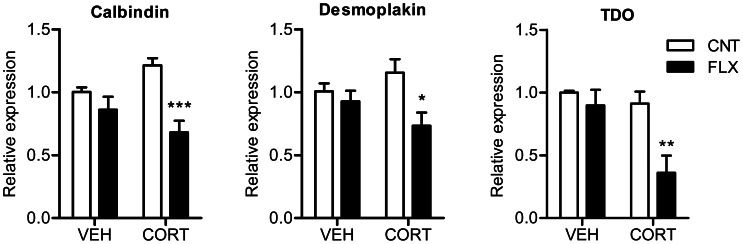
Corticosterone facilitates effects of fluoxetine on expression of mature granule cell markers. Calbindin, FLX effect: *P* = 0.0009, CORT×FLX: *P* = 0.0257. Desmoplakin, FLX effect: *P* = 0.0186. TDO, CORT effect: *P* = 0.011, FLX effect: *P* = 0.0086 (*n* = 4 each). *P<0.05, **P<0.01, ***P<0.001 compared with CNT/CORT.

The significant reduction of frequency facilitation by fluoxetine in corticosterone-treated mice may represent a change in the state of maturation of the dentate granule cells as shown in our previous study using high-dose fluoxetine [5]. To test this possibility, we examined expression of mature granule cell markers, calbindin, desmoplakin, and TDO [5], [15], [16], by using quantitative RT-PCR. Fluoxetine significantly reduced expression levels of all these maturation markers in corticosterone-treated mice, but not in vehicle-treated mice (Figure 4). There was significant interaction between corticosterone and fluoxetine treatments for calbindin expression (*P* = 0.0257). This result supports the idea that fluoxetine at 10 mg/kg/day induced granule cell dematuration in corticosterone-treated mice.

## Discussion

The present study has demonstrated that chronic corticosterone treatment facilitates the effects of fluoxetine on the gene expression in the dentate granule cells and on the dentate-to-CA3 signal transmission via the mossy fiber. In corticosterone-treated mice, fluoxetine at 10 mg/kg/day attenuated frequency facilitation at the mossy fiber synapse and downregulated the expression of molecular markers for mature granule cells. These changes are two major characteristics of the granule cell dematuration demonstrated previously in naive mice [5]. Therefore, these results suggest that corticosterone can facilitate the induction of granule cell dematuration by fluoxetine. Corticosterone also enhanced the augmentation of dopaminergic synaptic modulation by fluoxetine. The facilitated effect of fluoxetine is unlikely to be caused by altered drug metabolism, since there was no significant change in plasma concentrations of fluoxetine or its active metabolite norfluoxetine. Although we did not measure fluoxetine levels in the brain, a previous study reported no effect of corticosterone treatment on brain fluoxetine levels in a similar experimental condition [12]. This study by David et al. [12] examined stimulation of adult neurogenesis in the dentate gyrus by fluoxetine and also observed a facilitated effect of fluoxetine in corticosterone-treated mice. The central serotonergic system is essential for the granule cell dematuration and enhancement of monoaminergic modulation at the mossy fiber synapse by fluoxetine [5], [7] and for adult neurogenesis in the dentate gyrus [17]. Corticosterone locally infused into the hippocampus increases extracellular serotonin levels [18]. Chronic corticosterone treatment can attenuate 5-HT_1A_ autoreceptor-mediated inhibitory regulation of serotonergic neurons [19], which could lead to enhanced serotonin release in the target regions of serotonergic projections. Therefore, corticosterone may facilitate effects of fluoxetine via augmentation of the serotonergic transmission. Since corticosterone itself tended to change synaptic facilitation and dopaminergic modulation in the same direction as fluoxetine, fluoxetine and corticosterone may synergistically modify mossy fiber synaptic transmission and its modulation via activation of the serotonergic system. However, while fluoxetine had no effect on paired-pulse facilitation at an interval of 50 ms at 10 mg/kg/day (Figure 2D, see also [4]) and even at a high dose sufficient for the granule cell dematuration in naive mice (data not shown), corticosterone has significantly reduced this form of synaptic facilitation. Thus, the effects of fluoxetine and corticosterone on the mossy fiber synaptic facilitation appear to be mechanistically different. At vertebrate central synapses including the mossy fiber synapse, presynaptic calcium transients or currents are facilitated by the paired stimulation with short inter-pulse intervals [20], [21], [22], which can account for paired-pulse facilitation of synaptic transmission at least in part. Corticosterone may modulate this facilitation of presynaptic calcium transients, thereby attenuating paired-pulse facilitation at the 50-ms interval. At the calyx of Held, facilitation of presynaptic calcium currents can be induced at inter-pulse intervals shorter than 100 ms and declines during repetitive stimulation [20]. Therefore, the facilitation of calcium transients is less likely to be involved in frequency facilitation at 1 Hz and 0.2 Hz. Corticosterone has been shown to play an essential role in maintaining the anatomical and morphological integrity of the dentate gyrus and mossy fiber synapse [23]. Both lack and excess of corticosterone can impair the mature structure of the mossy fiber synapse [24], [25], suggesting requirement of optimal concentrations of corticosterone for maintaining the structure. It is possible that corticosterone is similarly involved in the maintenance of the functional maturity of the granule cell and/or mossy fiber synapse, and that excess corticosterone destabilizes its matured state, thereby facilitating dematuration by fluoxetine.

Adult neurogenesis in the dentate gyrus is suppressed and enhanced by corticosterone and SSRIs, respectively [26], [27], and SSRIs can reverse corticosterone-induced suppression of adult neurogenesis [11], [28]. Similar opposite effects of corticosterone and SSRIs have also been demonstrated for cell proliferation in the subventricular zone [29] and mRNA expression of brain-derived neurotrophic factor [30]. These previous results suggest that SSRIs could improve dysfunction of the central nervous system associated with chronic neuroendocrine disturbance in mood disorders. On the other hand, corticosterone and fluoxetine have been reported to additively downregulate 5-HT_1A_ autoreceptors in serotonergic neurons [19]. Our present study also showed that corticosterone tends to modify synaptic facilitation and dopaminergic modulation in the same direction as fluoxetine. Therefore, SSRIs may mimic or augment adaptive changes in the central nervous system associated with the neuroendocrine dysregulation. In corticosterone-treated mice, the dose of fluoxetine required for granule cell dematuration and marked enhancement of monoaminergic modulation was much lower than that in our previous studies using naive mice [5], [7] and fell within the range of the proposed optimal dose for mice [9]. The plasma level of fluoxetine was comparable to that of patients taking 80 mg/day fluoxetine, although the total levels of fluoxetine and norfluoxetine were higher in our study by about 70% [31], most likely due to differences in drug metabolism between humans and mice. Therefore, in certain pathological conditions, fluoxetine could induce granule cell dematuration and monoaminergic hyperfunctions near clinically relevant drug levels. In humans, the therapeutic efficacy of antidepressants including SSRIs has been shown to depend on the initial severity of depression [32], [33], [34]. Substantial antidepressant effects can be seen in patients with very severe depression, but there are minimal or no benefits over placebo in patients with mild or moderate depression [34]. The severity of depression has been shown to correlate with serum cortisol levels in the dexamethasone suppression test that assesses HPA axis dysregulation [35]. Therefore, the neuroendocrine state could be one of factors determining antidepressant responsiveness. Our present finding may explain such a difference in antidepressant efficacy associated with the state of the neuroendocrine system.

In conclusion, chronic corticosterone facilitates fluoxetine-induced neuronal plasticity in the dentate granule cells. In corticosterone-treated mice, the granule cell dematuration can be induced by fluoxetine at the dose much lower than that required in naive mice. Our present finding may provide insight into the neuronal basis for enhanced responsiveness to antidepressant medication in humans in certain pathological conditions.

## References

[1] Sahay A, Hen R (2007) Adult hippocampal neurogenesis in depression. Nat Neurosci 10: 1110–1115. 17726477 10.1038/nn1969

[2] Kobayashi K (2009) Targeting the hippocampal mossy fiber synapse for the treatment of psychiatric disorders. Mol Neurobiol 39: 24–36. 19130314 10.1007/s12035-008-8049-5

[3] Hanson ND, Owens MJ, Nemeroff CB (2011) Depression, antidepressants, and neurogenesis: a critical reappraisal. Neuropsychopharmacology 36: 2589–2602. 21937982 10.1038/npp.2011.220 PMC3230505

[4] Kobayashi K, Ikeda Y, Haneda E, Suzuki H (2008) Chronic fluoxetine bidirectionally modulates potentiating effects of serotonin on the hippocampal mossy fiber synaptic transmission. J Neurosci 28: 6272–6280. 18550770 10.1523/JNEUROSCI.1656-08.2008 PMC6670533

[5] Kobayashi K, Ikeda Y, Sakai A, Yamasaki N, Haneda E, et al (2010) Reversal of hippocampal neuronal maturation by serotonergic antidepressants. Proc Natl Acad Sci USA 107: 8434–8439. 20404165 10.1073/pnas.0912690107 PMC2889553

[6] Kobayashi K, Ikeda Y, Suzuki H (2011) Behavioral destabilization induced by the selective serotonin reuptake inhibitor fluoxetine. Mol Brain 4: 12. 21410937 10.1186/1756-6606-4-12 PMC3065414

[7] Kobayashi K, Haneda E, Higuchi M, Suhara T, Suzuki H (2012) Chronic fluoxetine selectively upregulates dopamine D_1_-like receptors in the hippocampus. Neuropsychopharmacology 37: 1500–1508. 22278095 10.1038/npp.2011.335 PMC3327854

[8] Kobayashi K, Manabe T, Takahashi T (1996) Presynaptic long-term depression at the hippocampal mossy fiber-CA3 synapse. Science 273: 648–650. 8662556 10.1126/science.273.5275.648

[9] Dulawa SC, Holick KA, Gundersen B, Hen R (2004) Effects of chronic fluoxetine in animal models of anxiety and depression. Neuropsychopharmacology 29: 1321–1330. 15085085 10.1038/sj.npp.1300433

[10] Parker KJ, Schatzberg AF, Lyons DM (2003) Neuroendocrine aspects of hypercortisolism in major depression. Horm Behav 43: 60–66. 12614635 10.1016/s0018-506x(02)00016-8

[11] Murray F, Smith DW, Hutson PH (2008) Chronic low dose corticosterone exposure decreased hippocampal cell proliferation, volume and induced anxiety and depression like behaviours in mice. Eur J Pharmacol 583: 115–127. 18289522 10.1016/j.ejphar.2008.01.014

[12] David DJ, Samuels BA, Rainer Q, Wang JW, Marsteller D, et al (2009) Neurogenesis-dependent and -independent effects of fluoxetine in an animal model of anxiety/depression. Neuron 62: 479–493. 19477151 10.1016/j.neuron.2009.04.017 PMC2759281

[13] Alvarez JC, Bothua D, Collignon I, Advenier C, Spreux-Varoquaux O (1998) Determination of fluoxetine and its metabolite norfluoxetine in serum and brain areas using high-performance liquid chromatography with ultraviolet detection. J Chromatogr B Biomed Sci Appl 707: 175–180. 9613947 10.1016/s0378-4347(97)00588-4

[14] Kobayashi K, Suzuki H (2007) Dopamine selectively potentiates hippocampal mossy fibre to CA3 synaptic transmission. Neuropharmacology 52: 552–561. 17049952 10.1016/j.neuropharm.2006.08.026

[15] Yamasaki N, Maekawa M, Kobayashi K, Kajii Y, Maeda J, et al (2008) Alpha-CaMKII deficiency causes immature dentate gyrus, a novel candidate endophenotype of psychiatric disorders. Mol Brain 1: 6. 18803808 10.1186/1756-6606-1-6 PMC2562999

[16] Ohira K, Hagihara H, Toyama K, Takao K, Kanai M, et al (2010) Expression of tryptophan 2,3-dioxygenase in mature granule cells of the adult mouse dentate gyrus. Mol Brain 3: 26. 20815922 10.1186/1756-6606-3-26 PMC2945337

[17] Brezun JM, Daszuta A (1999) Depletion in serotonin decreases neurogenesis in the dentate gyrus and the subventricular zone of adult rats. Neuroscience 89: 999–1002. 10362289 10.1016/s0306-4522(98)00693-9

[18] Barr JL, Forster GL (2011) Serotonergic neurotransmission in the ventral hippocampus is enhanced by corticosterone and altered by chronic amphetamine treatment. Neuroscience 182: 105–114. 21420472 10.1016/j.neuroscience.2011.03.020 PMC3091827

[19] Rainer Q, Nguyen HT, Quesseveur G, Gardier AM, David DJ, et al (2012) Functional status of somatodendritic serotonin 1A autoreceptor after long-term treatment with fluoxetine in a mouse model of anxiety/depression based on repeated corticosterone administration. Mol Pharmacol 81: 106–112. 22031471 10.1124/mol.111.075796

[20] Cuttle MF, Tsujimoto T, Forsythe ID, Takahashi T (1998) Facilitation of the presynaptic calcium current at an auditory synapse in rat brainstem. J Physiol 512: 723–729. 9769416 10.1111/j.1469-7793.1998.723bd.x PMC2231247

[21] Mori-Kawakami F, Kobayashi K, Takahashi T (2003) Developmental decrease in synaptic facilitation at the mouse hippocampal mossy fibre synapse. J Physiol 553: 37–48. 12963803 10.1113/jphysiol.2003.045948 PMC2343498

[22] Catterall WA, Few AP (2008) Calcium channel regulation and presynaptic plasticity. Neuron 59: 882–901. 18817729 10.1016/j.neuron.2008.09.005

[23] Kobayashi K (2010) Hippocampal mossy fiber synaptic transmission and its modulation. Vitam Horm 82: 65–85. 20472133 10.1016/S0083-6729(10)82004-7

[24] Sousa N, Lukoyanov NV, Madeira MD, Almeida OFX, Paula-Barbosa MM (2000) Reorganization of the morphology of hippocampal neurites and synapses after stress-induced damage correlates with behavioral improvement. Neuroscience 97: 253–266. 10799757 10.1016/s0306-4522(00)00050-6

[25] Sousa N, Madeira MD, Paula-Barbosa MM (1999) Corticosterone replacement restores normal morphological features to the hippocampal dendrites, axons and synapses of adrenalectomized rats. J Neurocytol 28: 541–558. 10800204 10.1023/a:1007015321767

[26] Cameron HA, Gould E (1994) Adult neurogenesis is regulated by adrenal steroids in the dentate gyrus. Neuroscience 61: 203–209. 7969902 10.1016/0306-4522(94)90224-0

[27] Malberg JE, Eisch AJ, Nestler EJ, Duman RS (2000) Chronic antidepressant treatment increases neurogenesis in adult rat hippocampus. J Neurosci 20: 9104–9110. 11124987 10.1523/JNEUROSCI.20-24-09104.2000 PMC6773038

[28] Qiu G, Helmeste DM, Samaranayake AN, Lau WM, Lee TMC, et al (2007) Modulation of the suppressive effect of corticosterone on adult rat hippocampal cell proliferation by paroxetine. Neurosci Bull 23: 131–136. 17612590 10.1007/s12264-007-0019-9 PMC5550626

[29] Lau WM, Qiu G, Helmeste DM, Lee TMC, Tang SW, et al (2007) Corticosteroid decreases subventricular zone cell proliferation, which could be reversed by paroxetine. Restor Neurol Neurosci 25: 17–23. 17473392

[30] Dwivedi Y, Rizavi HS, Pandey GN (2006) Antidepressants reverse corticosterone-mediated decrease in brain-derived neurotrophic factor expression: differential regulation of specific exons by antidepressants and corticosterone. Neuroscience 139: 1017–1029. 16500030 10.1016/j.neuroscience.2005.12.058 PMC1513636

[31] Pato MT, Murphy DL, DeVane CL (1991) Sustained plasma concentrations of fluoxetine and/or norfluoxetine four and eight weeks after fluoxetine discontinuation. J Clin Psychopharmacol 11: 224–225. 1741813 10.1097/00004714-199106000-00024

[32] Khan A, Leventhal RM, Khan SR, Brown WA (2002) Severity of depression and response to antidepressants and placebo: an analysis of the Food and Drug Administration database. J Clin Psychopharmacol 22: 40–45. 11799341 10.1097/00004714-200202000-00007

[33] Kirsch I, Deacon BJ, Huedo-Medina TB, Scoboria A, Moore TJ, et al (2008) Initial severity and antidepressant benefits: a meta-analysis of data submitted to the Food and Drug Administration. PLoS Med 5: e45. 18303940 10.1371/journal.pmed.0050045 PMC2253608

[34] Fournier JC, DeRubeis RJ, Hollon SD, Dimidjian S, Amsterdam JD, et al (2010) Antidepressant drug effects and depression severity: a patient-level meta-analysis. JAMA 303: 47–53. 20051569 10.1001/jama.2009.1943 PMC3712503

[35] Dratcu L, Calil HM (1989) The dexamethasone suppression test: its relationship to diagnoses, severity of depression and response to treatment. Prog Neuropsychopharmacol Biol Psychiatry 13: 99–117. 2748868 10.1016/0278-5846(89)90007-9

